# Entourage: all-in-one sequence analysis software for genome assembly, virus detection, virus discovery, and intrasample variation profiling

**DOI:** 10.1186/s12859-024-05846-y

**Published:** 2024-06-24

**Authors:** Worakorn Phumiphanjarphak, Pakorn Aiewsakun

**Affiliations:** 1 Department of Microbiology, Faculty of Science, Mahidol University, Ratchathewi District, 272 Rama VI Road, Bangkok, 10400 Thailand; 2 Pornchai Matangkasombut Center for Microbial Genomics, Department of Microbiology, Faculty of Science, Mahidol University, Bangkok, Thailand

**Keywords:** Virome, Metagenome, Virus detection, Virus discovery, Intrasample variation, Bioinformatics pipeline

## Abstract

**Background:**

Pan-virus detection, and virome investigation in general, can be challenging, mainly due to the lack of universally conserved genetic elements in viruses. Metagenomic next-generation sequencing can offer a promising solution to this problem by providing an unbiased overview of the microbial community, enabling detection of any viruses without prior target selection. However, a major challenge in utilising metagenomic next-generation sequencing for virome investigation is that data analysis can be highly complex, involving numerous data processing steps.

**Results:**

Here, we present Entourage to address this challenge. Entourage enables short-read sequence assembly, viral sequence search with or without reference virus targets using contig-based approaches, and intrasample sequence variation quantification. Several workflows are implemented in Entourage to facilitate end-to-end virus sequence detection analysis through a single command line, from read cleaning, sequence assembly, to virus sequence searching. The results generated are comprehensive, allowing for thorough quality control, reliability assessment, and interpretation. We illustrate Entourage's utility as a streamlined workflow for virus detection by employing it to comprehensively search for target virus sequences and beyond in raw sequence read data generated from HeLa cell culture samples spiked with viruses. Furthermore, we showcase its flexibility and performance on a real-world dataset by analysing a preassembled Tara Oceans dataset. Overall, our results show that Entourage performs well even with low virus sequencing depth in single digits, and it can be used to discover novel viruses effectively. Additionally, by using sequence data generated from a patient with chronic SARS-CoV-2 infection, we demonstrate Entourage's capability to quantify virus intrasample genetic variations, and generate publication-quality figures illustrating the results.

**Conclusions:**

Entourage is an all-in-one, versatile, and streamlined bioinformatics software for virome investigation, developed with a focus on ease of use. Entourage is available at https://codeberg.org/CENMIG/Entourage under the MIT license.

**Supplementary Information:**

The online version contains supplementary material available at 10.1186/s12859-024-05846-y.

## Background

Pan-viral metagenomic analysis remains a challenging problem. Unlike cellular organisms, viruses lack universally conserved genetic elements akin to 16S rRNA in bacteria [1], or to the nuclear ribosomal internal transcribed spacer region in fungi [2], that can serve as universal biomarkers for virus taxonomic identification and detection. Even viral proteins that are typically considered as highly conserved, such as the viral hallmark capsid proteins and RNA-dependent RNA polymerases, could still exhibit significant diversities at deep levels of evolutionary relatedness. For example, detecting similarities among capsid proteins from different viral orders or among RNA-dependent RNA polymerases from different viral phyla often necessitates three-dimensional structural analysis [3–5]. These examples highlight the immense diversity of viruses on the largest scale. Due to this, conventional virus detection methods, such as polymerase chain reaction (PCR) and Sanger sequencing, which heavily rely on high nucleotide similarity, can therefore have limited sensitivity for virus discovery, capable of detecting only viruses that the methods are designed for, or at least are very closely related to them.

Metagenomic next-generation sequencing (mNGS) offers a promising solution to overcome these challenges. This approach typically employs high-throughput 2nd generation sequencing technologies to sequence the entirety of DNA and/or RNA genetic materials in the analysed sample, generating millions of ‘short reads’ in an unbiased manner [6]. Unlike PCR and Sanger sequencing, mNGS can provide a comprehensive and unbiased overview of the microbial community, enabling detection of any viruses without the need for prior target selection. Another advantage of mNGS is its capacity to unbiasedly capture sequence variations within a single sample. While accurately determining minor variant frequencies could often be challenging [7], overall intrasample sequence variation profiles yielded from mNGS analysis have nevertheless proven valuable in, for example, studying virus microevolution [8], detecting subpopulations of drug-resistant viruses in individual patients [9, 10], and estimating the durations of viral infections [11].

One key challenge in utilising mNGS for virome investigation is that data analysis can be highly complex, involving numerous data processing steps. To address this challenge, we present Entourage, a comprehensive, versatile, and streamlined data analysis program for virome investigation. Entourage is developed with an emphasis on ease of use, and offers a wide range of sequence analysis functionalities, including read quality control and assembly, contig-based target virus detection, sensitive discovery of viral sequences, and intrasample genetic variation profiling. The program allows for multiple entry and end points in sequence analysis, accommodating various analytical needs, and generates comprehensive results that are straightforward to interpret, facilitating result quality control, reliability assessment, interpretation, and further downstream sequence analyses.

## Methods and materials

### Overview of entourage

Entourage is an open-source command-line program designed to facilitate and streamline the analysis of short-read assembly, contig-based virus identification and discovery, and quantification of intrasample sequence variations. The program is built using an *‘entourage*’ of well-received, well-established, open-source bioinformatics tools, and operates on the Linux operating system. Entourage uses Python3 for output processing, and Snakemake [12] for pipeline management, enabling sequential execution and parallelisation. Users can adjust the settings of all methods and software dependencies through a single well-structured configuration file, facilitating adjustability, transferability, and reproducibility. All data processing steps are logged, and reported with their execution times and parameters used, ensuring complete traceability of the program’s behaviours.

**Fig. 1:**
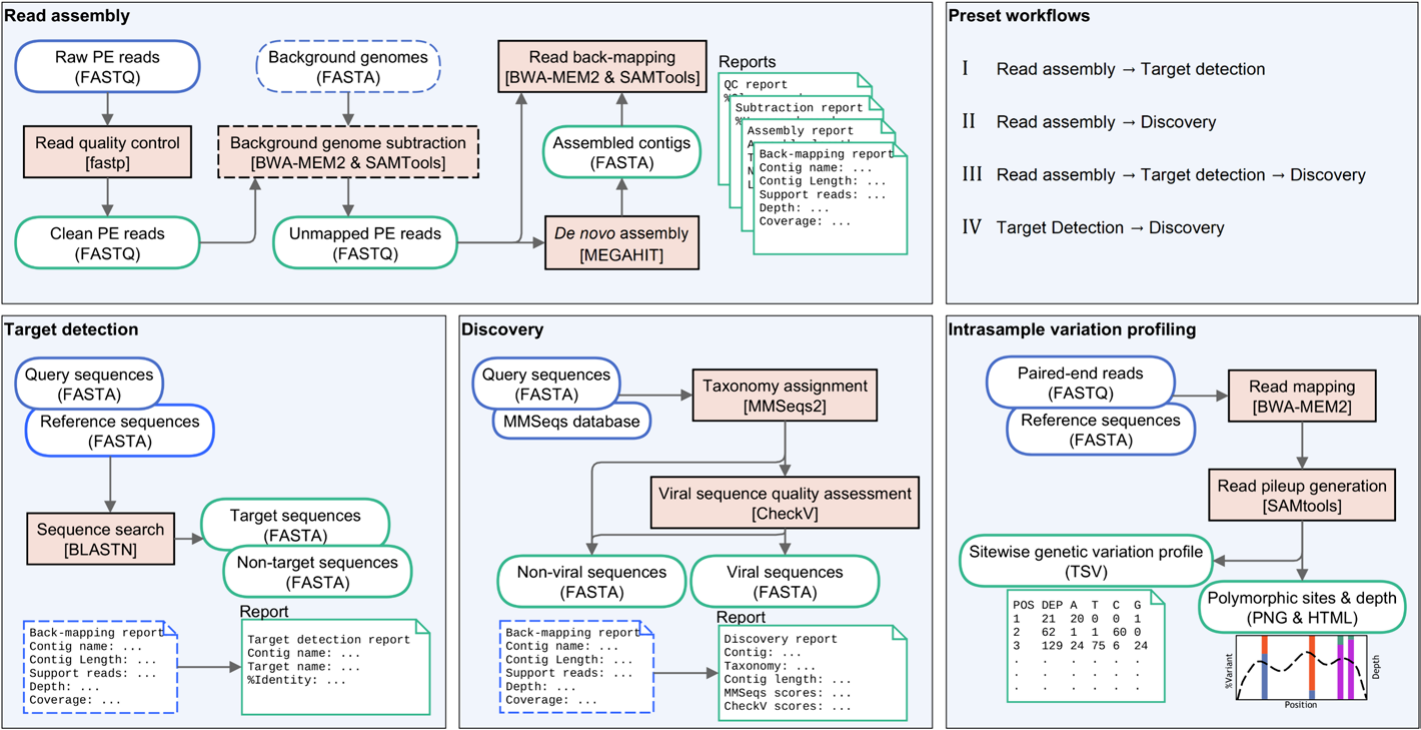
Overview of the four sequence analysis modules implemented in Entourage. Input files are indicated by blue outlines, while output files are indicated by green outlines. Sequence analysis processes are indicated by orange rectangles with the names of the program involved shown in square brackets. Optional inputs and processes are indicated by dashed outlines. PE – paired-end

Entourage offers four main sequence analysis functionalities, organised into separate modules: (i) read assembly module, (ii) target detection module, (iii) discovery module, and (iv) intrasample variation profiling module (Fig. 1). By combining the first three modules in different ways, Entourage also provides four workflows for virus sequence detection, streamlining sequence analysis according to different needs with just a single command line (Fig. 1 top right panel). Detailed descriptions of each sequence analysis module and workflow are provided below. Table 1 shows an overview of Entourage’s functionalities compared to several other well-received, and well-established metagenomic programs currently available.

**Table 1:** Entourage’s features compared to other currently available virus discovery software. Blank cells indicate that the feature is not explicitly mentioned in the software manual

Feature	Entourage	VirusSeeker [13]	SURPI [14]	GATK PathSeq [15]	VirFind [16]	VIP [17]	Lazypipe [18]	Genome Detective [19]	VIRify [20]
*Functionalities*									
Read quality control	**✓**	**✓**	**✓**	**✓**	**✓**	**✓**	**✓**	**✓**	
Background sequence subtraction	**✓**	**✓**	**✓**	**✓**	**✓**	**✓**	**✓**		
Read binning		**✓**	**✓**	**✓**		**✓**		**✓**	
Sequence assembly:	**✓**	**✓**	**✓**		**✓**	**✓**	**✓**	**✓**	
pre-taxonomic identification	**✓**	**✓**	**✓**		**✓**		**✓**		
post-taxonomic identification						**✓**		**✓**	
Target detection	**✓**			**✓**					**✓**
Virus discovery	**✓**	**✓**	**✓**	**✓**	**✓**	**✓**	**✓**	**✓**	**✓**
Intrasample variation profiling	**✓**								
*Input flexibility*									
Use raw reads as input	**✓**	**✓**	**✓**	**✓**	**✓**	**✓**	**✓**	**✓**	
Use assembled sequence as input	**✓**							**✓**	**✓**
*Software execution*									
Batch processing	**✓**	**✓**							
Local execution	**✓**	**✓**	**✓**	**✓**		**✓**	**✓**		**✓**

### Read assembly module

The read assembly module enables de novo assembly from raw paired-end short reads using MEGAHIT [21], along with read cleaning with fastp [22], which has been shown to be 2–5 times faster than other similar tools such as Trimmomatic [23] or Cutadapt [24]. It also offers an option to subtract reads from a specified set of organisms using BWA-MEM2 [25] and SAMtools [26]. By using simulated viral metagenomes as benchmarking datasets, studies have shown that MEGAHIT tends to produce more fragmented assemblies compared to other popular short-read assemblers, such as IDBA-UD [27] and metaSPAdes [28], but it has a superior, or at least very similar, genome fraction recovery rate and a lower misassembly rate ([29], and Table S4 in [30]), and thus it was chosen here. The module also performs read back-mapping using BWA-MEM2 [25], and computes various useful assembly-related statistics using SAMtools coverage [26].

### Target detection module

This module is designed for high-specificity virus sequence detection with prior knowledge of potential viruses in the examined sample. By using BLASTN [31], this module searches for a predefined set of viruses within assembled sequences based on nucleotide sequence similarity, and assigns taxonomic groups to them based on their BLASTN top hit. Under default settings, the top hit alignment must cover at least 50% of the contig with a minimum percentage identity of 90% to be considered a positive hit.

To facilitate downstream analyses, this module generates two FASTA file outputs: one containing sequences with positive hits to target viral sequences, and another containing those without. Besides taxonomic assignments, this module additionally reports for each contig its length, and weighted average BLASTN-hit percentage identity (i.e. $\sum \left(hit\_length \times \% identity\right)/\sum \left(hit\_length\right)$), as a tab-delimited table. If Entourage is used to perform read assembly, the table will also include the number of supporting reads, back-mapping coverage, and average sequencing depth for each individual contig, aiding in result reliability assessment and interpretation.

### Discovery module

This module enables sensitive virus sequence detection without requiring prior knowledge, using an amino acid similarity-based searching method. Since protein sequences are generally more conserved than nucleotide sequences, this module offers increased sensitivity compared to the target detection module, enabling detection of sequences not only of known viruses, but also potentially novel viruses that are distantly related to the references used. The core search engine employed by this module is MMSeqs2 taxonomy [32], which has demonstrated superior speed compared to CAT [33], which uses DIAMOND [34] at its core. Briefly, the program identifies open reading frames in input sequences, translates them, compares them against a reference protein sequence database, and then assigns an overall taxonomic identity (of various ranks) to each input sequence using an approximate dual BLAST-based last common ancestor strategy. Users need to prepare their own MMSeq2 taxonomic reference database to use this module.

One notable feature of MMseq2 taxonomy is that if an input sequence shows comparable similarities (as measured by -log(E value)) to multiple reference sequences from multiple taxonomic groups, the program will assign it to the last common ancestor of that group. In addition to its speed, this capability makes MMSeq2 taxonomy attractive for virus discovery, as novel viruses can oftentimes show detectable similarities to multiple known reference viruses at low levels. In such cases, assigning them to high taxonomic ranks rather than simply to the taxonomic group of the best hit can be more appropriate.

After MMseq2 taxonomic assignment, the module selects contigs assigned under the virus domain “d_Viruses”, and computes their percentage genome completeness and quality score using CheckV [35]. Users need to prepare their own CheckV database for this. Sequence quality is divided into five tiers: “Complete”, “High-quality” (> 90% completeness), “Medium-quality” (50–90% completeness), “Low-quality” (< 50% completeness), and “Not-determined” (no match against any CheckV reference genome with high enough similarity and any viral hidden Markov models (HMMs)). To facilitate downstream analyses, the program sorts viral contigs of different CheckV quality tiers into separate output FASTA files. Those that are annotated as non-viral or cannot be assigned into any taxonomic group by MMSeqs2 taxonomy are sorted into another separate sequence file. Similar to the target detection module, in addition to the CheckV quality score, percentage genome completeness, and contig length, if Entourage is used for read assembly, this module also reports information on the number of supporting reads, back-mapping coverage, and average sequencing depth for each contig.

### Intrasample variation profiling module

This module offers a streamlined procedure for computing sequence variations of a predefined set of viruses known to be present within the analysed sample. First, the module maps input sequence reads onto the reference sequences using BWA-MEM2 [25] to create a read alignment, and then uses SAMtools mpileup [26] to create a read pileup. Key default settings affecting BWA-MEM2 read mapping reliability and behaviours include (i) a seed length of 19, (ii) seed occurrences limited to 100, and (iii) ignoring reads with unmapped pairs. For SAMtools mpileup, these include (i) discarding anomalous read pairs, (ii) a minimum read mapping quality of 30, (iii) a minimum base quality of 30, and (iv) exclusion of reads flagged as “UPMAP”, “SECONDARY”, “QCFAIL”, or “DUP”.

The results are then parsed into a tab-delimited table, which can be analysed more readily compared to a Variant Calling Format file typically produced by standard variant callers. For each site, the results include the reference base, its overall sequencing depth, mean Phred base quality score, mean Phred read mapping quality score, forward and reverse read counts supporting A, T, C, G, deletion, and insertion variants, overall strand bias Χ^2^ test results, frequencies of each variant, and cumulative frequency of non-major variants. For sites with multiple nucleotide variants and when variant calling is supported by both forward and reverse reads, the Χ^2^ test determines if the variant profiles supported by forward and reverse reads are significantly different. Otherwise, i.e. when there is only one sequence variant, or when sequencing information comes from either forward or reverse reads, the test reports if the distribution of the overall forward and reverse read counts are significantly different from a uniform distribution. Regarding insertion variants, forward and reverse read counts of individual distinct insertion sequences are also reported, and following the convention, their overall frequencies are computed using the depth of the base position on their 5’ end. For deletion variants, depths of each individual deleted site are reported, computed based on reads spanning the deleted regions. This approach differs from those employed by many variant callers (e.g. LoFreq [36], and iVar [37]), which typically report depths of deletion regions only at their first 5’ end position.

To facilitate result interpretation, the module also generates a separate table containing just reliably detected polymorphic sites, which under default settings, are sites with (i) a mean Phred base score of ≥ 30, (ii) a mean Phred read mapping quality of ≥ 30, (iii) a sequencing depth of ≥ 100 × , (iv) at least one minor allele supported by ≥ 10 reads, and (v) a cumulative “supported” minor allele frequency of ≥ 5%. In addition, it produces a png figure illustrating the distribution of detected polymorphic sites, which can be readily used for publication. An interactive HTML version of the figure is also generated using the Plotly library [38] to facilitate detailed result inspection.

### Four virus sequence detection analysis workflows

Entourage offers four end-to-end workflows designed to streamline virus sequence detection analysis with one command line (Fig. 1 top right panel). One combines the read assembly module with the target detection module, facilitating the direct identification of a predefined group of target viruses from mNGS data. Another workflow combines the read assembly module and the discovery module together, allowing users to perform a more sensitive search for viral sequences directly from mNGS data without a predefined list of target viruses. The program also has a workflow that joins the read assembly module, the target detection module, and the discovery module together, enabling users to comprehensively search for both target viruses and beyond directly from their mNGS data. In addition, for uses with pre-assembled sequences, the program provides a workflow to search for target viral sequences and beyond within their assembled sequences as well. These workflows simplify the process of virus sequence detection, and at the same time offer flexibility to accommodate various analytical needs.

### Demonstration datasets

We illustrated the utility of Entourage for virus sequence detection using two publicly available mNGS datasets.

One dataset was raw sequence reads generated from HeLa cell culture samples spiked with four viruses, including Epstein-Barr virus (EBV), human respiratory syncytial virus (RSV), feline leukemia virus (FeLV), and human reovirus type 1 (REO1), published by Khan et al*.* [39] (Table S1). The viruses were spiked at concentrations of either 3 or 0.1 genome copies per HeLa cell (referred to as 3 × and 0.1 × samples, respectively). Both DNA and RNA sequences were combined for the analysis demonstration. HeLa cells are known to harbour 10–50 subgenomic sequences of human papillomavirus type 18 (HPV18) [40]; thus HPV18 sequences were also expected to be detected in this dataset with high sequencing depths. This dataset was used to demonstrate the read assembly—target detection—discovery workflow.

The second dataset was preassembled mNGS contigs generated from four marine samples subjected to different filtration schemes; all were collected during the Tara Oceans expedition at Station 125 in the South Pacific Ocean at 3–7 m below the surface level (Table S1). A previous analysis revealed that these samples are complex, containing diverse phages [20]. This dataset was used to demonstrate Entourage's capability to analyse preassembled sequences and to test the performance of the program, particularly the discovery module, against real-world data.

To demonstrate the functionality of Entourage’s intrasample variation profiling module, we used it to analyse sequence data from a coronavirus disease 2019 (COVID-19) patient with chronic severe acute respiratory syndrome coronavirus 2 (SARS-CoV-2) infection (sample P01A0207) published by Wang et al*.* [41]. Read cleaning and removal of human and phiX174 reads were performed using Entourage’s read assembly module. SARS-CoV-2 contigs were identified by comparing the assembled sequences against the reference Wuhan-Hu-1 SARS-CoV-2 genome (accession number NC_045512.2) using BLASTN. We found that the methods implemented in Entourage successfully assembled a full-length complete genome of the virus, covering > 99% of the reference sequence. “N”s were added to the beginning and the end of the assembly to adjust the base positions to match those of the reference Wuhan-Hu-1 SARS-CoV-2 genome. To ensure that the assembly was correct, we remapped clean high-quality reads to the assembly using BWA-MEM2 [25], used SAMtools [26] to remove reads with mapping scores ≤ 50 from the alignment, and created the major-variant sequence with iVAR [37] (i.e. having allele frequencies at all sites ≥ 50%). This sequence assembly and the clean reads were then analysed using the intrasample variation profiling module to detect polymorphic sites under default settings.

### Performance benchmarking

The performance of Entourage in virus sequence detection was benchmarked against two popular pipelines, including Lazypipe [18] and VIRify [20], using Khan et al.’s dataset. Both programs, like Entourage, offer a single command-line workflow for virus sequence detection, but their detection methods differ significantly.

Briefly, Lazypipe can perform read cleaning, background read subtraction, read assembly, and virus sequence annotation (Table 1). For this analysis, Lazypipe v3.0 was used. MEGAHIT was selected for sequence assembly, and the pre-set strategy “vi.nt” was employed to detect virus sequences (i.e., virus sequences were identified and annotated by mapping the assembled contigs to viral sequences from the NCBI nt database using minimap2, and BLASTN); other options used were default options. Unexpected viral sequences detected by the program (lengths ≥ 400 bases) were validated using reciprocal BLASTN analyses against the entire NCBI nt database.

VIRify, on the other hand, only offers virus sequence discovery in assembled sequence data (Table 1). The program uses VirFinder [42], VirSorter [43], and PPR-Meta [44], to initially predict virus contigs, and then annotates the predicted virus contigs using a database of virus protein HMM profiles, ViPhOGs [20]. The program subsequently assigns the annotated contigs to a taxonomy group that has at least 2 ViPhOG hits, with ≥ 60% of all ViPhOG hits linked to that one particular taxon [20]. Here, VIRify v2.0.0 was used with default settings to analyse the pre-assembled contigs generated by the Entourage read assembly module.

### Computational setup

Khan et al.’s dataset was analysed by Entourage, Lazypipe, and VIRify using a standalone computer running Ubuntu 20.04.2 LTS with 16 cores and 128 GB of memory available (see the Results section for the computational resources used). For the analysis of the Tara Oceans preassembled data and the demonstration of the intrasample variation profiling, we used a high-performance computing system running Ubuntu 20.04.2 LTS under the Slurm Workload Manager with 64 cores and 512 GB of memory. The analysis of the Tara Oceans preassembled data by the discovery module took 14.40 h to complete. For the demonstration of the intrasample variation profiling, the read assembly module took 0.07 h, and the intrasample variation profiling module took 0.10 h.

## Results

### Demonstration of virus sequence detection with the read assembly—target detection—discovery workflow

To demonstrate Entourage’s capability as a comprehensive workflow for virus sequence detection, we applied the read assembly—target detection—discovery workflow to analyse an mNGS dataset generated from human HeLa cell culture samples spiked with four viruses (EBV, RSV, FeLV, and REO1) at concentrations of 3 and 0.1 genome copies per HeLa cell (designated as 3 × and 0.1 × samples, respectively) published by Khan et al*.* [39]. Since HeLa cells are known to harbour HPV18 sequences [40], HPV18 sequences were thus expected to be found within this dataset as well. The reference database for the target detection analysis included 6560 “complete genomes” or “complete sequences” of the five expected viruses obtained from the NCBI nt database (EBV: 482 sequences, RSV: 5,126, FeLV: 24, REO1: 601, and HPV18: 327; Table S2). For the subsequent discovery analysis, the entire NCBI nr database was used as the reference database.

The read assembly module was used to clean the raw reads, subtract human and phiX174 reads, and generate assembled contigs. For each contig, the module reported its length, the number of supporting reads, back-mapping coverage, and average sequencing depth, facilitating assessment of read assembly quality (Supplementary File 1). Overall, the analysis revealed that < 0.5% of the high-quality reads were non-human / non-phiX174, and the program generated a total of 6,706 contigs with a minimum length of 400 bases from these reads across the two samples under default settings.

The target detection module successfully detected all five expected viruses in the 3 × sample, identifying 25 contigs as targeted viral sequences, showing percentage identities against target reference best hits between 93.64 and 100.00% (Table 2). In addition to taxonomic assignments, the raw outputs from this module included various statistics (see Methods), facilitating result reliability assessment and interpretation (Supplementary File 1). Some of the outputs are described and discussed below.

**Table 2:** Summary of the target detection module outputs from Khan et al*.*’s dataset analysis.

Virus	Overall sequencing depth ( ×)†	# of contigs	Contig lengths (bases)	Contig supporting reads	Contig sequencing depths ( ×)	% identities against target reference best hits
*3 × sample*						
EBV	16.22	7	2349–42,533	495–6558	15.53–105.03	99.25–100.00
RSV	57.59	1	15,407	8673	55.80	99.97
FeLV	6.21	3	649–4093	56–357	8.37–8.72	97.17–100.00
REO1 (11 segments)	51.98* (8.49–77.90)	11	407–3920	19–2697	4.69–77.83	93.64–100.00
HPV18	3,651.84	3	1053–2761	2130–144,366	201.84–5782.25	99.96–100.00
*0.1 × sample*						
EBV	0.45	3	427- 977	12–44	2.80–5.21	100.00–100.00
RSV	4.38	7	407–1176	13–180	3.23–17.93	99.77–100.00
FeLV	0.55	–	–	–	–	–
REO1 (11 segments)	0.47* (0.05–0.82)	–	–	–	–	–
HPV18	7,888.71	2	1262–5868	7514–402,649	596.71–6844.17	99.96–100.00

See Supplementary File 1 for raw outputs

^*^length weighted average. See sequencing depth of each individual segment in Table S3

^†^estimated mapping clean non-human / non-phiX174 reads against selected reference sequences (Table S3)

For the four spiked viruses, the numbers of supporting reads and depths of the detected contigs ranged between 19–8,673 reads and 4.69–105.03 × , respectively, while those of HPV18 were between 2,130–144,366 reads and 201.84–5,782.25 × , respectively. These much greater sequencing depths of HPV18 sequences were expected given that they are endogenous virus sequences in the HeLa cells. The overall virus sequencing depths estimated using a direct read-mapping approach ranged between 6.21 and 57.59 × for the four spiked viruses, and 3651.84 × for HPV18 (Table S3) falling within the range of individual contigs’ sequencing depths reported by Entourage. Altogether, these results support that the methods implemented in Entourage are sensitive, and still remain effective even when the average sequencing depth is in single digits.

For the 0.1 × sample, the module reported the detection of EBV (overall sequencing depth = 0.45 ×), RSV (4.38 ×), and HPV18 (7,888.71 ×) sequences, but did not detect FeLV (0.55 ×) and REO1 (0.05–0.82 ×) sequences (Table 2, Table S3, and Supplementary File 1). These findings revealed a decline in the performance of the methods used by our program when the sequencing depth is lower than 1 × . Indeed, genome assembly under such conditions is highly challenging, expected to produce highly fragmented and short contigs, explaining the results.

In this workflow, contigs not identified as target virus sequences are then passed to the discovery module. Briefly, the discovery module determines the taxonomic identity of input sequences using MMseq2 taxonomy [32], and computes the percentage genome completeness and quality score of each sequence annotated as viral by using CheckV [35], as well as reports various assembly-related summary statistics if the user employs Entourage for read assembly, allowing for thorough result assessment and examination. The outputs are summarised and discussed below.

Following the target detection analysis, the discovery module identified 3 and 9 additional contigs as viral sequences in the 0.1 × and 3 × samples, respectively, each containing at least one CheckV viral protein-coding sequence. Among these, one was annotated as a REO1 sequence and one as a HPV18 sequence (Table 3, and Supplementary File 1). Further investigation revealed that the REO1 sequence was missed by the target detection module as it showed only 89% identity to the best-hit target reference, falling below the default 90% threshold. For the HPV18 sequence, its target reference best-hit showed less than 50% contig coverage, and thus it was not reported by the target detection module; however, we found that the rest of the sequence actually also showed high similarity to some papillomavirus sequences in the NCBI database, but they were not included in the target reference database as their sequence records were not annotated as “complete genomes” or “complete sequences”. The remaining 10 contigs were identified as short sequences of phages (401–1173 bases), with supporting reads and sequencing depths ranging between 9 and 50 reads, and 1.99–4.73 × , respectively. Reciprocal BLASTN analyses against the entire NCBI nt database gave results consistent with those produced by Entourage (Table 3). Combined, these sequences were thus likely contaminated phage sequences in the samples. These results showcase the utility and high-sensitivity and specificity of our pipeline for virus discovery, as well as highlight the utilisation of both the target and discovery modules to maximise virus sequence detection capability.

**Table 3:** Summary of the discovery module outputs from Khan et al*.*﻿’s dataset analysis.

Contig	Taxon assigned by Entourage discovery module	Contig length (bases)	Contig supporting reads	Contig sequencing depths ( ×)	CheckV genome quality	% CheckV genome completeness	BLASTN best hit organism (acc number)	Sequence % identity	Contig % coverage
khan_3x_contig0093	Propionibacterium phage MEAK	444	12	2.66	Low-quality	1.48	Propionibacterium phage MEAK (MF919522.1)	94.81	99
khan_3x_contig3267	Orthoreovirus	2297	1383	60.45	Low-quality	9.82	Mammalian orthoreovirus 1 (AF174382.1)	99.95	99
khan_3x_contig4022	Pahexavirus	457	9	1.99	Low-quality	1.54	Caudoviricetes sp.(BK038489.1)	93.36	94
khan_01x_contig0259	Caudoviricetes	401	15	3.68	Low-quality	1.36	Propionibacterium phage PHL095N00 (NC_027401.1)	98.75	100
khan_01x_contig0278	Caudoviricetes sp.	611	25	4.08	Low-quality	2.07	Propionibacterium phage PHL112N00 (NC_022334.1)	97.22	100
khan_01x_contig0307	Propionibacterium phage PacnesP1	525	20	3.82	Low-quality	1.78	Propionibacterium phage PHL095N00 (NC_027401.1)	94.77	98
khan_01x_contig1506	Pahexavirus	497	15	3.04	Low-quality	1.69	Caudoviricetes sp. (BK048405.1)	98.36	98
khan_01x_contig2619	Caudoviricetes	719	34	4.73	Low-quality	2.38	Propionibacterium phage PHL009M11 (NC_027336.1)	94.43	99
khan_01x_contig3549	Caudoviricetes sp.	1173	50	4.27	Low-quality	3.98	Siphoviridae sp. ctkV91 (BK032807.1)	94.63	100
khan_01x_contig5237	human papillomavirus 18	901	8101	894.90	Low-quality	11.16	human papillomavirus 18 (MN164461.1)	100.00	96
khan_01x_contig5485	unclassified Caudoviricetes	486	16	3.32	Low-quality	0.58	Propionibacterium phage PHL025M00 (NC_027357.1)	95.27	100
khan_01x_contig5542	Propionibacterium phage Cota	407	11	2.64	Low-quality	1.38	Propionibacterium phage Pirate (NC_027623.2)	94.84	100

See Supplementary File 1 for raw outputs. Results from reciprocal BLASTN analysis with the entire NCBI nt database are also shown

Compared to two highly popular virus sequence detection pipelines, including Lazypipe [18] and VIRify [20], we found that Entourage is equally, if not more, sensitive in detecting virus sequences. For the 3 × sample, both Entourage and Lazypipe accurately detected sequences of all five expected viruses at the species level, whereas VIRify only detected sequences of EBV and RSV at the genus level, and sequences of REO1 at the subfamily level, but did not detect sequences of FeLV and HPV18 (Table 4). For the 0.1 × sample, while Entourage detected EBV, RSV, and HPV18 sequences, Lazypipe detected only EBV and RSV sequences, and VIRify did not detect any viral sequences (Table 4).

**Table 4:** Performance comparison of Entourage, Lazypipe, and VIRify for virus detection in Khan et al.’s dataset

	Entourage		Lazypipe		VIRify	
	3 × sample	0.1 × sample	3 × sample	0.1 × sample	3 × sample	0.1 × sample
Number of groups of expected viruses detected^*^	5	3	5	2	3^†^	–
Number of groups of unexpected viruses detected	2	6	5	8	–	–
To﻿tal time (hours)	15.06		﻿16.75		0.92‡	
Maximum memory usage (GB)	116.00		73.02		﻿1.61	

^*^ Expected viruses – EBV, RSV, FeLV, REO1, HPV18

^†^ VIRify identifies viral sequences at the genus level and above

^‡^ VIRify does not include read cleaning and de novo assembly steps

In addition, while VIRify did not report any unexpected viruses in this dataset, Lazypipe detected some unexpected viral sequences in both the 3 × and 0.1 × samples, similar to Entourage, including one *Orthopneumovirus bovis* sequence (which belongs to the same genus as RSV) and several other viral sequences (Table 4, and S4). However, unlike the results generated by Entourage (Table 3), none of these results by Lazypipe were corroborated by reciprocal BLASTN analyses, which instead suggested that most of them were bacterial sequences, and the one *Orthopneumovirus bovis* sequence was more likely a misidentified RSV sequence (Table S4). These results indicated that Entourage outperforms Lazypipe in terms of taxonomic assignment specificity and accuracy.

Regarding computation resource requirement (evaluated on a standalone computer with 16 cores and 128 GB of memory, Table 4), Lazypipe required 16.75 h, while Entourage was slightly faster, completing the analysis in 15.06 h. VIRify, which only performed sequence identification and taxonomic assignment, finished in 0.92 h. Entourage had the largest memory footprint, reaching a maximum memory usage of 116.00 GB, followed by Lazypipe at 73.02 GB, and VIRify at 1.61 GB. Inspection of Entourage’s log files revealed that this large memory consumption was due to the discovery analysis, which employed the entire NCBI nr database as a reference. The read assembly and target detection modules, on the other hand, did not require large memory, utilising only 23.42 and 0.25 GB of memory, respectively.

### Virus sequence detection performance against a real-world dataset

Entourage is highly flexible, allowing for multiple entry points in sequence analysis, and can also be used to analyse preassembled sequence data. To demonstrate this capability and at the same time showcase the program’s performance against real-world data, we used the discovery module to analyse preassembled sequences from the Tara Oceans expedition, previously analysed by VIRify [20].

Of the 1,534,855 assembled sequences, the discovery module annotated 30,232 as viral sequences with a CheckV quality score of the “Low-quality” tier or higher (Table 5 and Supplementary File 2). Among these, 10 were determined as CheckV complete sequences, while 11, 64, and 30,147 sequences had CheckV quality scores of “High-quality”, “Medium-quality”, and “Low-quality”, respectively. Of these sequences, 17,836 sequences were classified into five distinct phyla, with most of the sequences classified to the *Uroviricota* phylum (n = 16,664). The remaining 12,396 sequences were classified as viral, but MMSeq2 taxonomy could not further classify them into any lower taxa. Entourage detected all viral taxa detected by VIRify, except for the *Drexlerviridae* family, and notably vastly broadened the diversity of detected viruses to include three additional phyla and five classes.

**Table 5:** Virus sequences detected in the Tara Oceans dataset

Taxon	Viral contigs detected by Entourage			VIRify
	CheckV quality score			
	Complete	High/medium/low quality	Total	
*Uroviricota*		16,664	16,664	
*Caudoviricetes*		16,664	16,664	290
*Crassvira﻿les*		4	4	
*Methanobavir﻿ales*		2	2	
*Anaerodivi﻿ridae*		2	2	
Unclassified order		16,658	16,658	
*Ackermannviridae*		1	1	
*Arenbergviridae*		5	5	
*﻿Assiduviridae*		1	1	
*Autographiviridae*		402	402	5
*﻿Demerecviridae*		15	15	
*﻿Drexlerviridae*				6
*﻿Herelleviridae*		5	5	25
*Kyanoviridae*		4165	4165	
*Pachyviridae*		1	1	
*﻿Salasmaviridae*		3	3	
*﻿Stanwilliamsviridae*		2	2	9
*﻿Straboviridae*		5	5	216
*﻿Zobellviridae*		18	18	
*Nucleocytoviricota*		1138	1138	
*Megaviricetes*		1094	1094	127
*Algavirales*		335	335	10
*Phycodnaviridae*		335	335	10
*Imitervirales*		703	703	207
*Allomimiviridae*		34	34	
*Mimiviridae*		366	366	107
*Schizomimiviridae*		277	277	
*Pimascovirales*		25	25	
*Iridoviridae*		16	16	
*Marseilleviridae*		9	9	
*Pokkesviricetes*		3	3	
*Chitovirales*		3	3	
*Poxviridae*		3	3	
*Phixviricota*	1	21	22	
*Malgrandaviricetes*	1	21	22	
*Petitvirales*	1	21	22	
*Microviridae*	1	21	22	
*Preplasmiviricota*		10	10	
*﻿Maveriviricetes*		8	8	
*﻿Priklausovirales*		8	8	
*Lavidaviridae*		8	8	
*﻿Polintoviricetes*		1	1	
*Orthopolintovirales*		1	1	
*﻿Adintoviridae*		1	1	
*﻿Tectiliviricetes*		1	1	
*﻿Vinavirales*		1	1	
*Corticoviridae*		1	1	
*Cressdnaviricota*	1	1	2	
Unclassified viruses	8	12,388	12,396	
Total	10	30,222	30,232	417

Supplementary File 2 shows the full report of discovered viruses by the discovery module

Among the sequences not previously reported in [20] were 10 sequences detected as complete viral sequences by our program, including one *Microvidae* virus sequence, one *Cressdnaviricota* virus sequences, and eight sequences of unclassified viruses (Table 5). Upon searching these sequences against the entire NCBI nr database using BLASTP, we found that six of them likely represent sequences of novel phages from various taxonomic groups, containing structural protein-coding regions showing relatively low to medium percentage identities to known phage’s proteins (Table 6). In addition to highlighting the program’s flexibility, these results further underscore Entourage’s capability to detect novel viruses.

**Table 6:** Ten complete viral sequences detected by Entourage’s discovery module in the Tara Oceans dataset

Sequence	Taxon assigned by Entourage discovery module	Protein length (aa)	BLASTP best hit protein	Protein identity (%)	Contig coverage (%)	Organism of the BLASTP best hit protein
CEVC01358167.1	Prokaryotic dsDNA virus sp.	136	hypothetical protein (QDP59218.1)	100	100	Prokaryotic dsDNA virus sp.
CEVA01064070.1	Prokaryotic dsDNA virus sp.	320	hypothetical protein (QDP53221.1)	100	100	Prokaryotic dsDNA virus sp.
CEUT01008241.1	Prokaryotic dsDNA virus sp.	953	hypothetical protein (QDP50869.1)	100	100	Prokaryotic dsDNA virus sp.
CEUT01067160.1	Prokaryotic dsDNA virus sp.	1620	hypothetical protein (QDP59032.1)	100	100	Prokaryotic dsDNA virus sp.
CEUT01145058.1	Unclassified virus	312	putative capsid protein (AXH74836.1)	63.18	91	Cressdnaviricota sp.
CEUT01007640.1	Unclassified virus	174	hypothetical protein (AGA18343.1)	61.14	100	Uncultured marine virus
CEVA01513457.1	Microviridae sp.	286	DNA pilot protein (AXL15467.1)	45.77	46	Microviridae sp.
CEUT01145857.1	Cressdnaviricota	361	putative capsid protein (AXH76634.1)	45.48	94	Circoviridae sp.
CEVA01237671.1	Circular genetic element sp.	137	hypothetical protein (AXH79062.1)	35.21	99	Circular genetic element sp.
CEUT01055600.1	Prokaryotic dsDNA virus sp.	2332	hypothetical protein (QDP64034.1)	32.77	99	Prokaryotic dsDNA virus sp.

### Polymorphic site detection with Entourage

As an example of profiling intrasample variation with Entourage, we analysed high-depth sequence data from a patient with chronic SARS-CoV-2 infection published by Wang et al*.* [41], which had been reported to have a relatively high level of intra-host sequence variation. Under the following criteria: (i) base quality > 20, (ii) sequencing depth of paired-end mapped reads ≥ 10, (iii) > 5 reads supporting the minor allele, (iv) minor allele frequency ≥ 5%, (v) strand bias ratio of reads with the minor allele and reads with major allele less than ten-fold, and (vi) having just one minor allele, they reported 7 polymorphic sites and 1 variant (but non-polymorphic) site with respect to the Wuhan-Hu-1 sequence.

It has been suggested that, precision in variant frequency estimation generally scales with sequencing depth, and to reliably estimate variant frequency, the overall sequencing depth of a polymorphic site should be 10 times the reciprocal of the variants’ frequency [45]. This can be mathematically translated to that minor variants should have at least $\left(1/f\times 10\right)\times f$ = 10 supporting reads to be considered well-supported; where $f$ is the variant frequency. As such, under default settings, our module reports polymorphic sites with a minimum base and read mapping quality score of 30, sequencing depth of 100 × , having at least one minor allele supported by at least 10 reads, and a cumulative minor allele frequency of 5% excluding unsupported minor alleles (i.e. having < 10 supporting reads). However, these thresholds can be adjusted to be more (or less) conservative to best suit specific analytical needs. In addition to these, various statistics related to base and read mapping quality, variant supporting read counts and frequencies, and strand bias are reported as a tab-delimited table to facilitate result assessment.

**Fig. 2:**
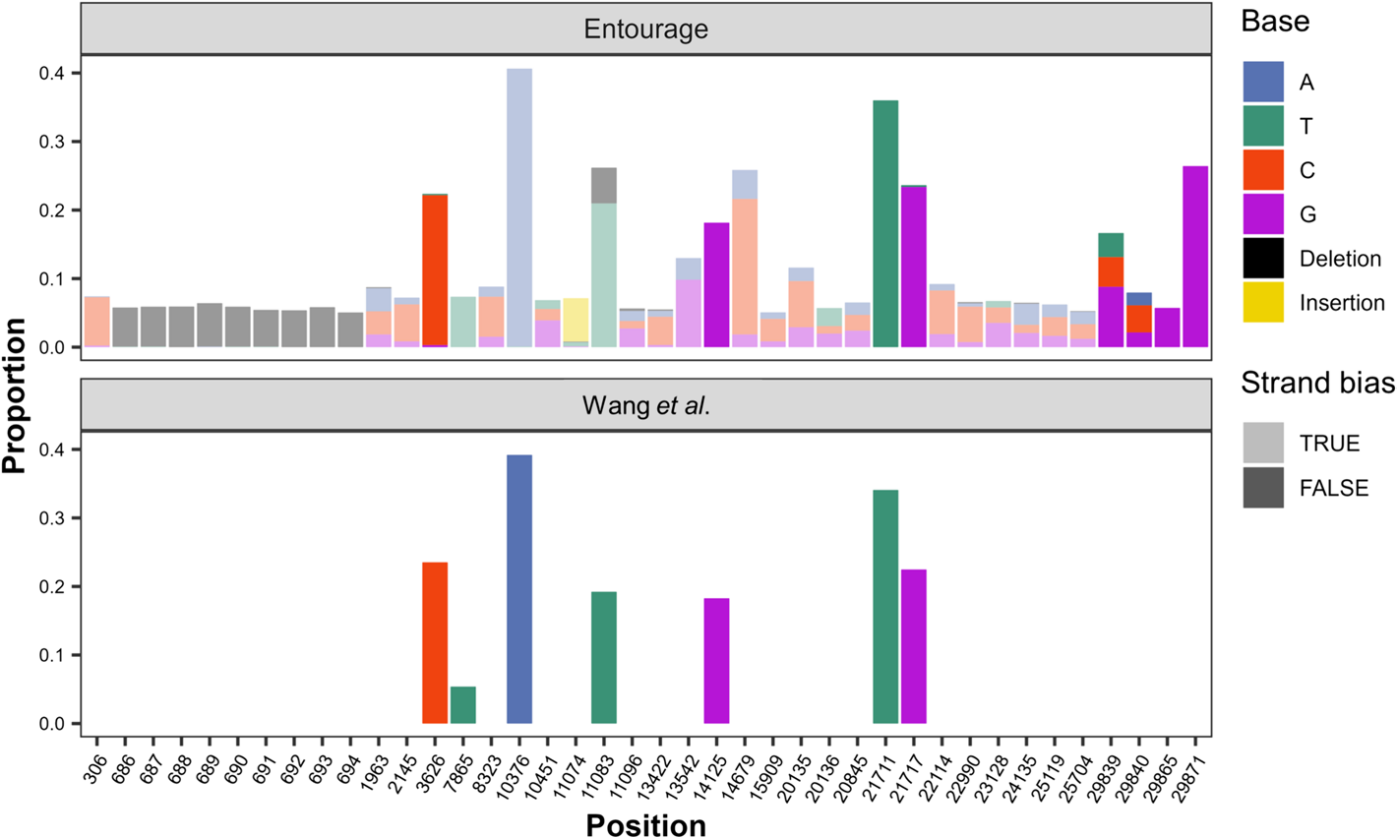
Polymorphic sites in SARS-CoV-2 from a chronic COVID-19 patient (sample P01A0207) published by Wang et al. [41]. Sites detected by our intrasample variant profiling module are shown at the top, and those reported by Wang et al*.* [41] are shown at the bottom. Lighter colours indicate strand bias. Only well-supported minor alleles (i.e., those with at least 10 supporting reads) are shown

**Fig. 3:**
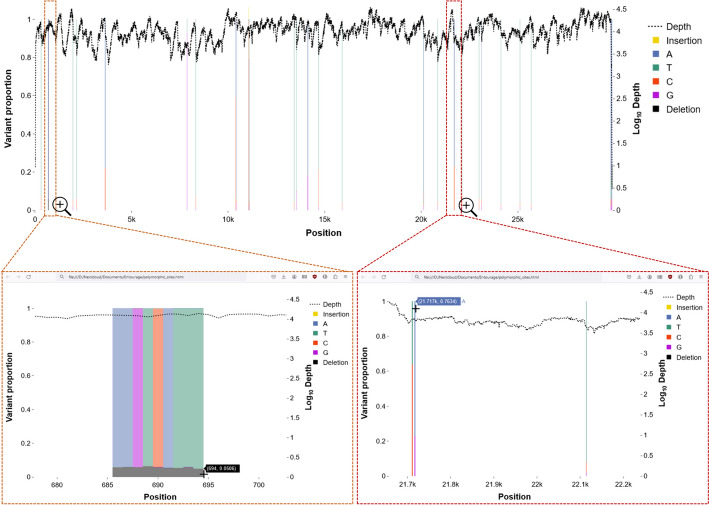
(Top) publication-ready png figure generated by Entourage for visualising the detected polymorphic sites, and (bottom) interactive HTML versions of the output, zoomed to show details of polymorphic sites at the positions 680–700 (left) and 21,700–22,200 (right). Vertical stacked colour bars indicate the detected polymorphic sites, with each colour corresponding to a different nucleotide variant, and lighter colours indicate strand bias. The height of each colour bar indicates the proportion of each nucleotide variant (left axis). The dotted line indicates the site-wise sequence depth on a logarithmic scale (right axis)

Using the major-variant sequence of the virus in the sample as the reference, Entourage detected 40 polymorphic sites in the virus (Fig. 2), with sequencing depths ranging between 159 and 22,682 × , mean Phred base quality scores ranging between 36 and 45, and all having mean Phred mapping quality scores of 60 (capped by BWA-MEM2). Based on Χ^2^ tests and Bonferroni multiple testing correction, Entourage reported that only 8 sites showed no overall strand bias (i.e., variant profiles supported by forward and reverse reads were not significantly different at the standard p-value threshold of 0.05). Figure 3 displays an example of a publication-ready figure generated by Entourage, along with interactive versions of the figure (Supplementary File 3) for magnifications of some specific positions. Supplementary File 4﻿ provides the raw outputs from the program and details of polymorphic sites identified by Entourage, enabling further detailed analyses and examination of the detected polymorphic sites if desired.

To highlight some key results, we found that Entourage successfully detected all 7 polymorphic sites reported by Wang et al*.* [41] with comparable minor allele frequency estimates (Fig. 2), and 3 of these sites showed strand bias under the Entourage default criteria. At site 11,083, besides the minor T allele variant (20.98%, 2,442 supporting reads) reported by Wang et al*.* [41], Entourage also identified an additional deletion variant (5.21%, 606 supporting reads) at this position*.* Moreover, Entourage detected 9 additional contiguous polymorphic sites (sites 686–694) characterised by having only one major minor deletion variant (5.06–6.33%, 703–724 supporting reads), which we therefore believe is a single contiguous 9-base deletion variant that was also previously overlooked by the original study. Sites 306, 29,865, and 29,871 were also identified by Entourage as polymorphic sites with only one major well-supported minor allele (Fig. 2), but their number of forward and reverse reads supporting their minor allele differed by more than 10 folds (Supplementary File 4), possibly explaining why they were not reported by Wang et al*.* [41]. The remaining polymorphic sites showed multiple alternative alleles, explaining the remaining differences in our results. These results illustrate how Entourage outputs can facilitate detailed result inspection.

## Discussion

Metagenomic sequencing is a powerful tool for studying viromes, but sequence data analysis can be complex. Here, we introduce Entourage, a comprehensive all-in-one bioinformatics tool for short-read sequence assembly, contig-based virus sequence detection, and intrasample sequence variation profiling (Fig. 1, and Table 1). The program is versatile, enabling both hypothesis-driven virus sequence detection with the target detection module, and exploratory virus discovery analysis with the discovery module. Entourage offers multiple entry points to accommodate different input data types, and provides several streamlined workflows for automated, end-to-end virus sequence detection analysis with different analytical needs. The results produced by the program are highly comprehensive, facilitating result quality control and reliability assessment. Additionally, Entourage supports batch processing, allowing efficient handling of a large number of datasets by automating data processing with preconfigured parameters.

Two distinct viral sequence detection modules are implemented in Entourage: the target detection module, and the discovery module. By using BLASTN [31] as a search engine, the target detection module offers a relatively faster search for viral sequences showing high nucleotide similarity to the user-defined target viral sequences. This module is particularly suitable for investigations where prior knowledge of the viruses present in the samples is available. The discovery module, on the other hand, allows for viral sequence discovery without prior knowledge of the viruses present in the sample. It uses MMSeqs2 taxonomy [32] as its core search engine, detecting viral sequences based on protein sequence similarity searching. Both modules require assembled sequences as input, necessitating users to perform sequence assembly, either by using the read assembly module provided within Entourage or alternative software tools. Although this prerequisite may impose greater computational time and resource demands compared to other programs, it is expected that this issue will become increasingly less of a concern as computer hardware and software continue to advance.

One notable advantage of the contig-based approach to virus sequence detection (as opposed to read-based approaches, see below) is that it can offer a relatively higher sensitivity for virus sequence detection, for instance, by enabling detection of nucleotide regions with low similarity to viruses in the reference database but of significant lengths. In addition, by examining assembled sequences, it becomes possible to identify complete open reading frames, and infer their corresponding full-length protein products. Since protein sequences are generally more conserved than nucleotide sequences, this method can potentially offer greater sensitive than nucleotide similarity searching, allowing for detection of viruses that are distantly related to those in the reference database. Indeed, this approach has facilitated the discovery of many novel viruses [46–48]. Moreover, various in-depth sequence analyses, such as detailed virus characterisation, exploration of genome structural variations, functional analysis, and phylogenetic studies, often necessitate viral sequence assemblies. Therefore, sequence assembly should not be regarded as excessive in this context, but as a crucial step that enhances virus detection, and at the same time facilitates comprehensive downstream analyses of the detected viruses.

To illustrate Entourage’s utility as a streamlined pipeline for virus sequence detection, we applied the read-assembly—target detection—discovery workflow to analyse raw mNGS data generated from HeLa cell culture samples spiked with viruses previously published by Khan et al*.* [39] (Tables 2 and 3, and Supplementary File 1). The results were compared against those generated by two popular virus discovery tools, including Lazypipe, and VIRify (Table 4, and S4). Also, to demonstrate the program’s flexibility and performance with real-world data, we applied the discovery module to search for virus sequences in a preassembled Tara Oceans dataset (Tables 5 and 6, and Supplementary File 2). Overall, our findings indicate that the methods implemented in Entourage work well for virus sequence detection, even when the virus sequencing depth may be in single digits, and they can be used to discover novel viruses effectively. By using Khan et al*.*’s dataset as a benchmarking dataset, we showed that Entourage has greater sensitive in detecting virus sequences than Lazypipe and VIRify especially those with low sequencing depths, with more accurate and precise taxonomic assignments overall (Table 4 and S4). In addition, we showcased how detailed outputs generated by Entourage can be meaningfully interpreted and are useful for result quality control and reliability assessment.

Of note, we observed a decline in Entourage’s performance for virus sequence detection when the overall virus sequencing depth dropped below 1 × . These results were perhaps anticipated, as genome assembly becomes highly challenging when certain parts of the genome regions are missing, resulting in highly fragmented and short contigs. In such cases, read-based methods may be more preferable. These methods might involve direct mapping of sequence reads to a diverse set of virus reference sequences (e.g., VIP [17], Genome Detective [19], and MetaShot [49]), or comparing the nucleotide *k*-mer profiles of sequence reads to a *k*-mer profile database generated from a wide array of sequences (e.g., Bracken [50], Kraken2 [51], and CLARK [52]). In addition, read sequences covering coding regions, in part or in whole, can be used for a more sensitive read-based search at the amino acid level (e.g. BLASTX [31], TBLASTX [31], DIAMOND [34], UBLAST/USEARCH [53], and Kaiju [54]). While it has been shown that read-based methods often fall short in virus discovery, for instance, an analysis of marine viromes reported that up to 91% of the metagenomic reads generated from marine samples could not be taxonomically identified based on sequence similarity to known taxa even when BLASTX was used [55], these methods may offer advantages when sequencing depths are very low.

We also observed that, with short reads, genome assemblies of some viruses, particularly those with large genomes, can still be fragmented even with adequate sequencing depth. For example, while our pipeline could recover 93% of the EBV genome from the spiked 3 × sample, its assembly was found to consist of 7 contigs instead of just one single contiguous sequence even with an overall sequencing depth of 16.22 × . This highlights a well-known limitation of short-read sequence de novo assembly, especially for large genomes with repetitive elements, and the importance of thorough result examination, and verification. Long-read sequencing and hybrid assembly may offer solutions to this problem, potentially allowing for more accurate and more contiguous assembled sequences. While Entourage currently does not support long-read and/or hybrid assembly, the target detection and discovery modules can be independently executed on assembled sequence data. Thus, users can assemble their own (long-read and short-read) sequences (should they have them) using other bioinformatics tools such as metaSPAdes [48], metaviralSPAdes [49], metaFlye [50], or viralFlye [51], and subsequently use our program to analyse the assembled sequences in FASTA format. This approach may help improve the recovery of large and more complete virus genomes.

In the broader context of virus discovery and detection, the choice of reference database can significantly impact result reliability. Horizontal gene transfers between viruses and cellular life forms are well documented [56, 57]. Also, while the process of mutation is random, natural selection is not, and thus, at least in theory, convergent evolution has the potential to cause non-viral sequences to look somewhat like viral sequences even in the absence of gene transfers (i.e. in this context, showing detectable sequence similarities and/or E-values that are much lower than 1 in sequence searches). As such, if the reference database contains just viral sequences, chances are that some non-viral sequences could be systematically incorrectly identified as viral sequences, especially with highly sensitive search engines. Indeed, our results revealed that, with a virus-only reference database, Lazypipe can sometimes misidentify bacterial sequences (as suggested by reciprocal BLASTN analyses against the entire NCBI nt database) as viral sequences (Table S4). Thus, with the discovery module, we recommend using the entire NCBI nr database as the reference database to minimise false positives, especially with diverse samples. Indeed, with this approach, we demonstrated that Entourage did not show these kinds of false-positive calls. In addition, since virus taxonomy is still not complete and is still evolving [58], and that the taxonomic assignments by MMSeq2 taxonomy relies heavily on the NCBI taxonomy tree, we recommend users to regularly update the tree of the MMSeq2 database to ensure accurate taxonomic assignments.

Analysis of mNGS data also enables intrasample variation profiling. We showed that Entourage can effectively quantify virus intrasample sequence variation. As shown in the Result section, this module generates comprehensive outputs that can help with result quality assessment and interpretation. In addition, to the best of our knowledge, Entourage is the first program to offer a single-step procedure form analysing mNGS data to produce intrasample variation profiles in a tabular format, detailing site-wise compositions of all bases and indels, to generating publication-quality figures illustrating the results. This information can reveal new mutations and/or coexistence of multiple virus variants, enabling one to gain a deeper insight into the dynamics of viral populations within a single sample.

### Availability and requirements

- **Project name**: Entourage
- **Project home page**: https://codeberg.org/CENMIG/Entourage
- **Operating system**: Linux
- **Programming language**: Bash and Python
- **Other requirements**: Python3.10, Snakemake, and other third-party software like fastp, MEGAHIT, BLAST, MMseq2, etc. Check the manual for a complete list (https://codeberg.org/CENMIG/Entourage#dependency-installation).
- **License**: MIT
- **Any restrictions to use by non-academics**: None

### Supplementary Information

Additional file1: Raw outputs generated by Entourage from the analysis of Khan et al.’s dataset, containing 6 tables. These include (i) read cleaning results, (ii) read subtraction results, (iii) read assembly statistics, (iv) read back-mapping results, (v) the reports from the target detection module, and (vi) the report from the discovery module.

Additional file2: Report file generated by Entourage from the analysis of the Tara Oceans dataset using the discovery module.

Additional file3: Interactive version of the polymorphic site graph (Figure 2) in HTML format generated by Entourage from the polymorphic site detection analysis of SARS-CoV-2 from a chronic COVID-19 patient (sample P01A0207) published by Wang et al.

Additional file4: Report outputs generated by Entourage’s intrasample variation profiling module from the analysis of SARS-CoV-2 from a chronic COVID-19 patient (sample P01A0207) published by Wang et al., containing 2 tables. These include (i) nucleotide variant profiles of all sites, and (ii) nucleotide variant profiles of polymorphic sites only.

Additional file5: Table S1 Dataset compositions and sources. Table S2 Reference sequences used in the analysis of Khan et al.’s dataset with the target detection module. Table S3 Average mapping coverages and depths of viruses in Khan et al.’s dataset. Table S4 Reciprocal BLASTN results for unexpected viral sequences detected in Khan et al.’s dataset by Lazypipe.

## Data Availability

The datasets analysed during the current study are available from the NCBI Sequence Read Archive database (See Table S1). Raw outputs from Entourage are provided as Supplementary Files.

## Abbreviations

PCR: Polymerase chain reaction
mNGS: Metagenomic next-generation sequencing
EBV: Epstein-Barr virus
RSV: Human respiratory syncytial virus
FeLV: Feline leukemia virus
REO1: Human reovirus type 1
HPV18: Human papillomavirus type 18
COVID-19: Coronavirus disease 2019
SARS-CoV-2: Severe acute respiratory syndrome coronavirus 2
HMM: Hidden Markov model
